# Qigong and Fibromyalgia: Randomized Controlled Trials and Beyond 

**DOI:** 10.1155/2014/379715

**Published:** 2014-11-12

**Authors:** Jana Sawynok, Mary Lynch

**Affiliations:** ^1^Department of Pharmacology, Dalhousie University, P.O. Box 15000, Halifax, NS, Canada B3H 4R2; ^2^Departments of Anesthesia, Psychiatry, and Pharmacology, Dickson Centre, QEII Health Sciences Centre, 5820 University Avenue, Halifax, NS, Canada B3H 1V7

## Abstract

*Introduction*. Qigong is currently considered as meditative movement, mindful exercise, or complementary exercise and is being explored for relief of symptoms in fibromyalgia.* Aim*. This narrative review summarizes randomized controlled trials, as well as additional studies, of qigong published to the end of 2013 and discusses relevant methodological issues.* Results*. Controlled trials indicate regular qigong practice (daily, 6–8 weeks) produces improvements in core domains for fibromyalgia (pain, sleep, impact, and physical and mental function) that are maintained at 4–6 months compared to wait-list subjects or baselines. Comparisons with active controls show little difference, but compared to baseline there are significant and comparable effects in both groups. Open-label studies provide information that supports benefit but remain exploratory. An extension trial and case studies involving extended practice (daily, 6–12 months) indicate marked benefits but are limited by the number of participants. Benefit appears to be related to amount of practice.* Conclusions*. There is considerable potential for qigong to be a useful complementary practice for the management of fibromyalgia. However, there are unique methodological challenges, and exploration of its clinical potential will need to focus on pragmatic issues and consider a spectrum of trial designs. Mechanistic considerations need to consider both system-wide and more specific effects.

## 1. Introduction

Fibromyalgia (FM) is characterized by widespread pain and multiple somatic symptoms. In the 1990s, the American College of Rheumatology (ACR) provided criteria involving presence of allodynia (tender point examination) and other symptoms including fatigue, sleep disruption, and gastrointestinal symptoms [1]. A recent PubMed search indicated 361 English language articles with “fibromyalgia” as a keyword in the 20-year period prior to the 1990 ACR criteria being published, and 3844 articles in the 20-year period following publication attesting to growing recognition of the condition [2]. In 2010, modified criteria were developed to address the issue of tender points, associated symptoms, and the observation that FM might represent an extreme end of a pain continuum; updated criteria include a chronic widespread pain index and symptom severity scale for cognitive symptoms, sleep disturbance, fatigue, and somatic symptoms [3]. The prevalence of FM is 0.5–5% while that of chronic widespread pain is 10–20% [2, 4]. FM is a challenging condition, both for those who experience it and for those who treat it [5, 6]. Longitudinal benefits are modest despite use of currently approved drugs [7, 8]. Mechanisms involved in FM include central sensitization, altered central pain processing, impaired endogenous pain regulation mechanisms, and disturbances in the hypothalamus-pituitary-adrenal axis, the autonomic nervous system, and peripheral tissues [2, 4]. Current treatment guidelines emphasize patient education, pharmacological and nonpharmacological approaches, and treatment of comorbid conditions [5, 9, 10].

Qigong (Chi Kung, Chi Gong) refers to cultivation (practice, discipline) of qi (life energy, energetic essence) and has a long history in China, extending thousands of years, as a health and wellness practice [11–13]. Many forms of qigong have developed, reflecting particular contexts for development (martial arts, health practice, and spiritual practice). The modern history of qigong began in China in the 1950s; western interest grew in the 1960s and has further accelerated in recent decades. As a self-practice, internal qigong involves dynamic (movement) and static (quiescent) elements and involves regulation of movement, breath, and awareness. When highly developed by skilled practitioners, qigong can be applied as external qigong, whereby the practitioner directs energy (using hand movements, focused attention) towards an individual to improve the flow of qi. In the past decade, qigong has been characterized as “mindful exercise” [14] or “meditative movement” [15], and these conceptualizations are useful for considering qigong in relation to other practices such as mindfulness, meditation, and conventional exercise.

There have been several recent (published 2012-2013) systematic reviews and meta-analyses of randomized controlled trials (RCTs) of qigong for fibromyalgia. Some consider qigong as a distinct entity [16, 17] while others consider it as a “meditative movement therapy” or “complementary and alternative exercise” whereby qigong, tai chi, and yoga are clustered together [18, 19]. There also have been reviews of qigong in the even broader context of complementary and alternative therapies [20, 21]. These overviews reach different conclusions regarding the potential of qigong for fibromyalgia, ranging from cautious (may be useful, but emphasize the limited quality of trials and methodological challenges) [16, 17] to indicating it may be a useful component of multimodal treatment [18, 19] and even to proposing a rational usage algorithm (for complementary therapies in general) [21].

In addition to RCTs, there is an increasing recognition of the need for pragmatic or effectiveness trials to provide information for improving the management of chronic pain [22]. Furthermore, multiple diverse trial designs need to be considered for exploring the effectiveness of complementary and alternative medicine (CAM) modalities [23–26]. Several trial variations are now available and provide additional insight into potential benefits of qigong. The purpose of the present narrative review paper is to describe (1) evidence for efficacy of qigong in FM (RCTs, reviews, and others) published to the end of 2013 and to discuss (2) the relationship between practice and outcomes, (3) methodological issues that require attention, and (4) potential mechanisms of action of qigong.

## 2. RCTS of Qigong for Fibromyalgia

There are 7 RCTs of qigong for FM published to the end of 2013 (Table 1). Six of these trials involved adults (*N* = 367, mean age 45–57 years, and mean FM duration 5–15 years) and one involved children and adolescents (*N* = 30, mean age 13 years). Three RCTs involved qigong instruction and daily home-practice over 6–8 weeks and monitored adherence to practice [27–29]. One trial over 7 weeks did not appear to require home-practice for the qigong group but did for the comparator group [30]. Two RCTs over 8–12 weeks involved weekly group sessions where qigong was part of a multimodal session (with meditation, body awareness, and education) and no home-practice was performed [31, 32].

Two RCTs (*N* = 57, *N* = 100) involving qigong (7-8 weeks, daily home practice) compared outcomes to wait-list subjects and reported significant benefits in pain, impact, sleep, physical and mental function, and quality of life following the intervention and at 4–6 months of follow-up [27, 29]. Post-intervention standard mean differences (SMD, or Cohen's d, is the difference between means divided by the pooled standard deviation (M1−M2)/√[*σ*_1_^2^ + *σ*_2_^2^]; values of ≥0.2 are small, ≥0.5 are medium, and ≥0.8 are of large effect sizes) across these domains were 0.44–0.73 [27] and 0.71–1.16 [29] compared to the wait-list group. At follow-up, there was some drift but significant effects were maintained (SMDs 0.2–0.7 in [27]; 0.52–0.72 in [29]). A smaller RCT (*N* = 14) of qigong (7 weeks, daily home practice) compared qigong to sham exercise and reported significant postintervention benefits in the qigong group but not the sham exercise group (effect sizes 0.99–1.98), but there was no follow-up [28]. One RCT (*N* = 30) compared qigong (QG) to another movement and body awareness method (Rességuier method, RM) and compared two groups in which these methods were used sequentially for 7 weeks (RM + QG, QG + RM). The first 7 weeks of this trial represents a comparative trial of QG versus RM. In both groups, there were significant and comparable postintervention improvements in pain, impact, mood, and function following the first intervention; addition of the second intervention did not lead to further improvement, but benefits were maintained at 6-7 months [30]. SMDs compared to baseline ranged from 0.7 to 1.5 postintervention and at follow-up.

Two earlier RCTs (*N* = 128, *N* = 38) of qigong involved weekly sessions (8–12 weeks, but no home practice) in which qigong was part of a multimodal approach; both noted no differences in pain or impact compared to the comparison group (education support, normal daily activity) [31, 32]. There were beneficial changes across time in both the intervention and the control groups in one study [31], but few differences in the other [32]. The RCT in children and adolescents (*N* = 30) compared qigong to aerobic exercise (3 sessions/week) and monitored many aspects of physical function and functional outcomes; benefits were reported in both groups, but the aerobics group performed better on several measures [33].

## 3. Other Trials of Qigong for Fibromyalgia

Additional information on the effects of qigong for FM is available from other trials that use heterogeneous approaches (Table 2). Three pilot studies examined qigong (all used different forms) in an open-label manner over 3–9 weeks with follow-up to 3–6 months. Creamer et al. [34] (*N* = 28) included qigong as one component of a multimodal intervention (weekly for 8 weeks, no home practice) and observed significant improvements in many areas that were maintained over time (4–6 months); however, it is not possible to discern an effect of qigong in this study due to the multimodal nature of the intervention (included educational, cognitive/behavioural, and relaxation/meditation elements, all of which potentially contribute to benefit). Chen et al. [36] (*N* = 13) used external qigong (5–7 sessions over 3 weeks) and observed significant pre-post intervention improvements (SMDs 0.7–1.9) that were maintained at follow-up. Two participants in this study had such marked reductions in symptoms that they were considered “cured” by the intervention, and their end symptomology was minimal. Lynch et al. [37] (*N* = 23) examined qigong practice over 9 weeks with daily home-practice and follow-up to 6 months; significant improvements in several areas were observed over time. The latter trial informed the conduct of a larger RCT (Table 1(6)). Attrition in the three pilot studies over 3–9 weeks was 23–39%.

Information on the effects of extended practice of qigong is available from case studies and an extension trial (Tables 2(4) and 2(5)). There are qualitative case reports of individuals (*N* = 2) who engage in extended qigong practice (≥1 hr/day) over longer periods of time (1-2 years) in a community setting which indicate marked reductions in FM symptoms (pain, sleep disturbance, impact, mood, and quality of life), as well as health improvements in other areas (allergies, vision, skin, and circulation) [38]. There is another case of extended qigong practice in a community setting providing marked health benefits in several areas [41]. Effects of long-term practice of qigong in FM also were addressed in an extension trial in which *N* = 20 who had completed an earlier controlled trial went on to a further 6-month phase (total practice ≥1 year) [39]. *N* = 13 completed the extension, and their outcomes indicate that extended qigong practice resulted in significant gains in core FM areas (pain, impact, sleep, and physical function and mental function) in quantitative assessments. In the *N* = 5 subgroup who had previously voluntarily undertaken additional practice prior to the extension and who practiced the most during the extension trial, end symptomology was mild, and qualitative comments indicated additional health benefits (food allergies, chemical sensitivities, asthma, migraines, blood pressure, and vision; some discontinued several medications). The other *N* = 8 who completed the extension phase had similar improvements in pain, impact, sleep, and physical and mental function in quantitative scores, but their end symptomology was higher (as were baselines). In addition, their qualitative comments were clearly more moderate in tone, and there was little mention of health benefits in other areas. It appears that a subgroup of individuals benefits greatly from extended qigong practice, with respect to both FM and other symptoms. The amount of practice undertaken by these individuals would be difficult to study in a prospective manner, and extension trial methodology is a useful and practical consideration for documenting effects. A qualitative post hoc analysis of comments of RCT participants who completed or did not complete the extension trial, and considered in relation to amount of practice, reveals that initial favourable experiences with the practice predispose to continued practice and better outcomes (Table 2(6)).

## 4. Methodological Issues

Qigong is a complex practice involving regulation of movement, breath, and attention, and methodological issues related to qigong studies have been considered recently [42]. Some of these relate to general experimental design (e.g., sample size, description of practice, blinding, and controls), while others relate to factors that are specific to qigong (e.g., diversity of interventions, differing doses and duration, and mixture of active factors). Tai chi research issues share many of these features [43, 44]. Methodological issues particularly relevant to qigong and FM are summarized in Table 3.

The following considerations relate to studies of qigong for FM summarized in Tables 1 and 2.

*(1) Plurality of Forms.* Studies are from several geographic locations (United States, Canada, Sweden, and Italy) and reflect different forms that are available locally. There is considerable heterogeneity in the amount of details provided as to the nature of practice in these studies. There are no comparative trials of different forms of qigong for FM, and it is not clear whether all forms of qigong would necessarily produce the same results. 

*(2) Components of Practice.*The recent designation of qigong as “meditative movement” and “mindful exercise” has been useful in terms of identifying core elements (deconstruction), using recognizable language to define instructions (operationalization), and providing relevant comparisons (qigong versus other movements or meditative practices); these facilitate the design, conduct, and interpretation of clinical trials into qigong. 

*(3) Amount of Practice.* Table 1 summarizes RCTs that examined qigong over 6–8 weeks and involve regular/daily home-practice and follow-up to 6 months (Tables 1(4)–1(7)). These trials uniformly report significant benefits in core FM domains (pain, sleep, impact, physical and mental function, and quality of life) compared to wait-list and/or significant and sustained improvements over time compared to baseline. Trials involving weekly sessions whereby qigong was part of a mixed session noted little difference or ambiguous results and are difficult to interpret specifically in relation to qigong (Tables 1(1) and 1(2)). One trial performed a post hoc analysis in relation to amount of qigong practice and noted significant differences in outcomes between those who practiced daily and those who practiced minimally (Table 1(6)). Table 2 provides further insight, noting case studies and extension trial participants who engage in extensive practice and report markedly improved health outcomes. Collectively, this information indicates that benefit is related to amount of practice. However, with increased practice time comes increased attrition, and the number who engaged in extended practice is limited. The factors that predispose to such extended practice are not clear, but post hoc analysis of the qualitative information from a RCT does indicate that good experiences over the initial 8 weeks of practice predispose to continued practice and better outcomes (Table 2(6)). 

*(4) Effectiveness of Practice.* Not all practice time is equally effective with meditative practices, and it can be a challenge to address this component. A meditative movement inventory [45] has been designed but is not validated or widely used. The mindfulness and body awareness literatures face a similar challenge and also are developing measures to address this issue [46, 47]. 

*(5) Control or Comparison Group.* RCTs in Table 1 use a variety of control groups, and each has its merits and limitations. It is important to distinguish between a group where the intent is to utilize a sham (presumed inert) procedure which lacks the active component and a group where the intent is to compare effects of qigong to another active group. With the latter approach, there may not be a difference between qigong and active comparators in between-group analyses. However, within-group analysis compared to baseline for both groups can be assessed and considered in relation to benchmark clinical outcomes, and this provides a further valuable perspective. 

*(6) Multiple Trial Designs.* RCTs and pragmatic trials for chronic pain have unique strengths and limitations and inform different contexts (regulatory processes, clinical care) [22]. Both approaches provide information on qigong for FM (Tables 1 and 2). Mixed-methods research that includes qualitative information is of further value, especially with extended practice. 

*(7) Participants.* The mean age of adult participants in qigong for FM trials ranges from 42 to 57 years and the mean FM duration ranges from 5 to 15 years, and these are typical of FM trials in general. One trial conducted a post hoc analysis of those with FM duration above and below the median of 9 years and observed no differences in pain and impact with qigong [29]. There is the potential to explore subgroups in a prospective manner in relation to factors relevant to self-practice (e.g., locus of control, motivation, and psychological characteristics). Furthermore, with a practice that is less familiar than other practices (exercise, meditation, and yoga), it may be important to determine attitudes towards complementary and alternative therapies and especially in relation to extended practice. 

*(8) Outcomes*. FM outcomes can align with general IMMPACT guidelines for chronic pain and FM-specific OMERACT guidelines [48, 49]. Between-group analysis of outcomes provides a statistical measure of effectiveness, but not necessarily a clinical assessment of effectiveness. Effect size analysis (standard mean difference, Cohen's d) provides an indication of whether an effect is small (≥0.2), medium (≥0.5), or large (≥0.8). Changes that constitute clinically important outcomes for pain (30–35% reduction from baseline) [50, 51] and impact (14% reduction from baseline) [52] have been determined and provide further benchmarks. These interpreted and benchmark parameters are of particular importance for within-group analyses.

## 5. Mechanisms of Qigong

Mechanistic considerations for qigong can be global (system-wide) or specific (focus on cellular, molecular, or chemical mediators). Within a traditional framework, there is a qi-matrix which interacts dynamically with physiological systems; qigong practice smooths and strengthens the circulation of qi within this system, and unrestricted qi flow leads to health and longevity [11, 12]. Contemporary language for this concept is coherence and resonance within a living system matrix which allows for self-organization in a complex system leading to integrated function [11, 53]. Qigong has also been characterized as a complex biopsychosocial activity [12, 14, 54]. Within this framework, psychological factors (e.g., cognitive/behavioral, social interactions) and physiological factors (e.g., cardiovascular fitness, autonomic nervous system regulation, central neurotransmitter systems, neuroendocrine, and stress hormone systems) contribute to mind/body integration and a state of health. A further conceptualization considers qigong as a complex intervention involving somatic regulatory systems and neurohormonal and neurotransmitter mediators; it considers movements in relation to metabolic expenditure, rhythm, and posture and reflects on interoception, imagery, and neuroplastic changes [42]. The latter acknowledges that a shortcoming of this area of investigation is a lack of taxonomy of components within recognizable categories.

Additional studies examine effects of qigong on specific functional parameters and biomarkers. An analysis of 26 trials published from 1997 to 2006 indicates that qigong has effects on immune function (white blood cells, lymphocytes), cardiovascular function (cardiac kinetics, blood pressure), and respiratory function (capacity, exchange) [55]. Other reviews note effects of qigong on biomarkers of stress, inflammation, and immune function [56, 57]. Such studies are limited by the multiple forms of practice in individual studies, variable durations of practice, and variable linkages with clinical outcomes.

Some recent intriguing studies demonstrate that extended qigong practice can lead to changes at a molecular level. Thus, there is a report that extensive qigong practice (1-2 hrs daily, for at least a year) leads to altered expression of 250 genes in neutrophils compared to healthy controls, with changes characterized by enhanced immunity, downregulation of cellular metabolism, and alteration in apoptotic genes in favor of resolution of inflammation [58]. However, that study was small and did not include functional effects. Another report on a therapeutic regimen of qigong (twice-weekly group practice for 5 weeks, daily home-practice for 12 weeks) compared to a wait-list control group reported significant beneficial effects in chronic fatigue syndrome (which exhibits overlap with FM [59, 60]) and demonstrated a significant increase in telomerase activity after 4 months of practice [61]. It will be important for future studies of qigong to examine functional outcomes and biomarkers particularly relevant to those conditions. It will also be important to consider the amount of practice time in a systematic manner in relation to these events.

## 6. Summary and Perspective

Complementary therapies represent a diverse group of therapies and practices and there are attempts being made to find their place in treatment of FM [20, 21]. Evidence-based guidelines from different countries differ in recommendations relating to complementary therapies [62]. The current review focuses on qigong, considers RCTs as well as other studies, and provides an additional perspective beyond a singular focus on RCTs. Certain themes are emerging. 

*(1) The Magnitude and Duration of Benefit Is Significant.* Regular self-practice (particularly daily) for 6–8 weeks leads to effect sizes of 0.5–0.8 and beyond for pain, sleep, impact, and physical and mental function, and benefits are maintained at follow-up at 4–6 months [27–30]. These effects are manifest when compared to wait-list subjects and/or baselines, and effects are consistently moderate-to-large over a range of core domains for FM. Other body awareness regimens practiced diligently (daily, for 7 weeks) produce similar effects to qigong [30]. While there can be concern over whether effects of qigong are specific or nonspecific, and this is relevant to regulatory and mechanistic considerations, the observation most relevant to clinical care is magnitude of post-intervention effects that are sustained over time. The magnitude of other nonpharmacological approaches can be similar to qigong, while pharmacological effects are generally modest [10, 63–65]. This emphasizes the need to more fully explore nonpharmacological modalities for FM. 

*(2) The Amount of Qigong Practice Matters.* There has been limited systematic assessment of the relationship of outcomes to amount of qigong practice, but several observations are relevant. Thus (a) RCTs that utilize limited amounts of qigong practice (weekly session) report more limited and ambiguous outcomes compared to those that engage in regular practice (6–8 weeks, daily practice); (b) outcomes are related to amount of practice within a trial (minimal practice versus daily practice); (c) extended practice (≥1 hr/day, ≥6–12 months) leads to marked health benefits in FM and other areas. Practice time and compliance will be important to consider in future controlled trials and in systematic reviews and meta-analyses of qigong. 

*(3) There Is a Need for Additional Exploration of Qigong.* The literature on qigong for FM is limited by the small number of RCTs and heterogeneity of qigong styles and study designs, but the magnitude, scope, and duration of beneficial effects in core domains of FM with regular qigong practice are intriguing and the practice merits further attention. The benefits of extended qigong practice are particularly notable, but there are few such reports. There is a particular need to further explore extended qigong practice in observational trials, both as extension trials to RCTs, and in those who practice in a community setting. Thus, a recent review of FM guidelines from several countries concluded by noting “FM cannot be cured by any therapy” [62] yet extended qigong practice has produced marked health benefits in FM in a subset of individuals, and their experiences can provide valuable insights. It is impossible to blind qigong practice, so it will be important to examine intact systems of practice. Additional studies need to make direct comparisons to other practices (exercise, other movements, and meditative methods) and with drug regimens, psychological methods (e.g., cognitive/behavioural therapy), and even combinations of such approaches (multimodal therapy). They also need to examine components of practice (deconstruct elements, work towards a more uniform and recognizable taxonomy) and address mechanistic substrates (at systems, cellular and molecular levels). Finally, barriers to the practice of qigong need to be identified (e.g., nature of instruction, conceptualization, language, optimal training regimes, motivating factors, and subgroup factors) as these can impact on our understanding of the health potential of this practice.

## Figures and Tables

**Table 1:** Summary of randomized controlled trials (RCTs) of qigong for fibromyalgia. Control groups include passive (normal activities, wait-list) and active groups (education, aerobic exercise, sham exercise, and other interventions).

Study Participants, study features	Intervention, duration, measures	Outcomes				
		Between group comparisons, except where indicated				
(1) Astin et al. 2003 [31] *N* = 128; QG + MM *N* = 64 CG (education support) *N* = 64 Mean age: 47.7 yrs Mean FM duration: 5.0 yrs Attrition: 50 (39%) by 8 wks, 61 (48%) by 16 wks, and 67 (49%) by 24 wks Note: The 1st controlled study of QG for fibromyalgia.	Intervention: QG (Dance of Phoenix), mindfulness meditation (MM); 2.5 hr total (QG = 60 min), weekly group session Duration: 8 wks Measures: BL, 8 wks; 4 and 6 mos F-UP		**Post-intervention**	**Follow-up**		
		FIQ	NS	NS		
		Total myalgic score	NS	NS		
		SF-36 pain	NS	NS		
		6 min walk test	NS	NS		
		Depression (BDI)	NS	NS		
		Note: (1) Due to high attrition, only those completing the entire protocol were analysed. (2) Within-group comparisons at 8 wks and 4 and 6 mos indicate that both groups had significant improvements over time in FIQ, total myalgic score, SF-36 pain, and depression; effects were manifest at 8 wks and maintained to 24 wks.				

(2) Mannerkorpi and Arndow 2004 [32] *N* = 38; QG + BA *N* = 19 CG (normal activities) *N* = 17 Mean age: 45 yrs Mean FM duration: 10 yrs Attrition: 14 (39%)	Intervention: QG (style not reported), body awareness (BA) therapy; 1.5 hr total (QG = 20 min), weekly group session Duration: 3 mos (14 sessions) Measures: BL, 3 mos Note: Participants were encouraged to practice at home, but none regularly performed home exercises.		**Post-intervention**			
		BARS	*P* < 0.05			
		FIQ	NS			
		Handgrip test	NS			
		Chair test	NS			
		Note: (1) Due to attrition, only 12 + 10 participated in post-test outcomes. (2) Within-group BARS analysis indicated significant changes in treatment group but not control group; within-group FIQ total score analysis indicates significant changes in control group but not treatment group.				

(3) Stephens et al. 2008 [33] *N* = 30; QG *N* = 16 CG (aerobic exercise) *N* = 14 Mean age: 13.3 yrs Attrition: 6 (20%) Note: The 1st controlled trial of QG as exercise intervention in children and adolescents with FM.	Intervention: QG exercises (18 postures, style not reported) versus aerobic exercise; 30 min of practice in 1 group session + 2 home sessions each week Duration: 3 mos Measures: BL, 3 mos Note: Adherence monitored by heart rate monitors and diary entries (noted as 60–70%)	**Post-intervention**, there were significant improvements in physical function, functional capacity, QOL, and fatigue in the aerobics group. Aerobic function, tender point count, pain, and symptom severity improved similarly in both groups; the aerobics group performed better in several measures compared to the QG group. Note: (1) The study addressed both physical fitness and clinical symptoms and monitored multiple outcomes. (2) Intent-to-treat analysis was used.				
	** **	** **	** **	** **		

(4) Haak and Scott 2008 [27] *N* = 57; QG *N* = 29 CG (wait-list) *N* = 27 Mean age: 53.3 yrs Mean FM duration: 15.4 yrs Attrition: 1 (2%) Note: At the end of the wait-list interval, subjects received QG training and constituted a delayed training cohort.	Intervention: QG (He Hua Qigong) 11.5 hrs instructions/practice over 7 wks; encouraged to practice at home (2 × 20 min/day); subjects also had 2 external QG sessions Duration: 7 wks Measures: BL, 8 wks, 4 mos F-UP Note: At postintervention, the combination group had high compliance with 93% reporting regular (3–6 days/wk) or daily practice. At 4 mos F-UP, 65% were still practicing and 90% of those on a regular-daily basis.		**Between-group**		**Within-group *P* values**	
		Pain (VNS)	**Cohen's *d***		**Pre-post**	**Pre-F-UP**
		Intensity	0.63		<0.001	<0.05
		Inconvenience	0.73		<0.01	<0.05
		Control of pain	0.61		<0.01	NS
		Sleep (VNS)				
		Restoration	0.44		<0.001	<0.01
		Quality	NS		<0.001	<0.01
		Psychological				
		STAI (anxiety)	0.66		<0.05	<0.01
		BDI (depression)	0.69		<0.01	<0.001
		QOL total	0.55		<0.001	<0.01
		QOL psychological	0.52		<0.001	<0.001
		QOL physical	NS		<0.01	<0.001
		Note: (1) Many between-group effect sizes following the intervention are in the moderate-to-large range (0.5–0.8). (2) Combination group data reports postintervention and longer-term outcomes for N = 56 subjects. (3) While benefits are sustained over time, SMDs at follow-up compared to BL range from 0.2 to 0.7.				

(5) Liu et al. 2012 [28] *N* = 14; QG *N* = 8 CG (sham exercise) *N* = 6 Mean age: 56.5 yrs Mean FM duration: 9.4 yrs Attrition: 12.5% (2/8) in intervention group Note: This is the only study to use sham exercise as a comparison.	Intervention: QG (Liu Zi Jue Qigong, “Six Healing Sounds”), 2 training sessions, weekly group sessions (45–60 min), daily home-practice (2 × 15–20 min, morning and evening). Sham had the same movements, but no meditation or healing sounds. Duration: 6 wks Measures: BL, 6 wks		**Pre-post QG**		**Pre-post Sham**	
		SF-MPQ (pain)	↓44% (*P* < 0.0125)		↓10% (NS)	
		MFI (fatigue)	↓25% (*P* < 0.0125)		↓6% (NS)	
		PSQI (sleep)	↓37% (*P* < 0.0125)		↓10% (NS)	
		FIQ (impact)	↓44% (*P* < 0.0125)		↓12% (NS)	
		Note: (1) Between-group Cohen's *d* for pain, fatigue, sleep, and impact was 1.56, 1.58, 0.99, and 1.98, respectively. (2) Trial is limited by the small sample size and lack of follow-up data.				
** **	Note: Participants asked to keep diary of home-practice. Compliance was moderately high (75–85% daily, 77–79% group sessions).	** **	** **	** **	** **	

(6) Lynch et al. 2012 [29] *N* = 100; QG *N* = 53 CG (wait-list) *N* = 47 Mean age: 52 yrs Mean FM duration: 9.6 yrs Attrition: 12% (12/100) at 6 mos; 29% (12/42) delayed intervention group Note: (1) The largest QG for FM trial to date. (2) At the end of the wait-list interval, subjects received QG training and constituted a delayed training cohort.	Intervention: QG (Chaoyi Fanhuan Qigong, CFQ); 3 half-day training sessions (4 hrs each); weekly review/practice session (60 min); daily home-practice (45 mins) Duration: 8 wks Measures: BL, 8 wks, 4 and 6 mos F-UP Note: (1) Compliance with practice was reported as average weekly practice time at 8 wks and at 4 and 6 mos F-UPs. (2) For all who completed the 6-month trial, 85% (62/73) practiced ≥ 3 hrs/week and 52% (38/73) ≥5 hrs/week at 8 wks.			**Post-QG**	**4 mo F-UP**	**6 mo F-UP**
		NRS (pain)	imm	<0.001	0.01	<0.05
			del	0.01	0.005	<0.05
		FIQ (impact)	imm	<0.001	0.003	0.007
			del	<0.001	0.01	0.05
		PSQI (sleep)	imm	0.001	<0.001	0.003
			del	0.009	NS^*^	NS^*^
		SF-36 physical	imm	<0.001	<0.001	0.004
			del	<0.01	<0.001	0.01
		SF-36 mental	imm	0.002	NS	NS
			del	0.004	NS	NS^*^
		Note: (1) Individual QG groups (imm, del) showed good reproducibility when compared to wait-list. (2)^*^P < 0.01 in combination group analysis. (3) Combination group data reports post-intervention and follow-up data for the N = 73 who completed the trial to 6 months; it includes participants from both the immediate and the delayed intervention groups. (4) Combination group SMD values compared to wait-list are 0.81 (0.67, 0.56 at F-UP) for pain, 1.16 (0.63, 0.57 at F-UP) for impact, 0.71 (0.60, 0.52 at F-UP) for sleep, 0.80 (0.72, 0.56 at F-UP) for physical function, and 0.79 (0.23, 0.49 at F-UP) for mental function. (5) In the *N* = 38 per protocol group (practiced ≥5 hrs/week at 8 wk), SMD values versus wait-list are pain 1.17 (0.96, 0.93 at F-UP), impact 1.67 (0.88, 0.88 at F-UP), sleep 1.04 (0.97, 0.71 at F-UP), physical function 0.95 (0.93, 0.79 at F-UP), and mental function 1.07 (0.39, 0.62 at F-UP).				
						
(7) Maddali Bongi et al. 2012 [30] *N* = 30 (RM + QG *N* = 15; QG + RM *N* = 15) CG (RM over the first 7 weeks) Mean age: 57.3 yrs Mean FM duration: 7.2 Attrition: 0% once training commenced (but 8/38 withdrew following randomization) Note: (1) Comparative trial between two active methods considered as “mind-body” practices. Both groups represent potentially active interventions. (2) Initial 7 weeks is comparison trial; the remainder of study represents a crossover trial but can also be considered an add-on trial.	Group1: RM + QG Group2: QG + RM 7 wks (1 wk break) + 7 wks 2 sessions/week for wks 1–3 1 session/week for wks 4–7 (total 10 sessions, 45–60 mins) RM: home exercise of 30 min/day during intervention Duration: 15 wks, F-UP 12 wks after completing the 2nd method Measures: BL, 7 wks (T1), 15 wks (T2), 27 wk F-UP Note: (1) Detailed description of QG and RM provided (QG style not specified). (2) RM practiced daily for 30 mins, but this was not specified for QG.		**Pre-post *P* values (within group)**			
		**Group 1: RM + QG**	**After RM**	**After QG**	**F-UP**	
		FIQ	<0.001	<0.001	<0.001	
		HAQ	<0.001	<0.001	<0.001	
		NRS (pain)	<0.001	<0.001	<0.001	
		RPS (pain)	<0.001	<0.001	<0.001	
		NRS (sleep)	NS	NS	NS	
		HADS-A	<0.001	<0.001	<0.001	
		HADS-D	NS	<0.001	<0.001	
		SF-36 PCS	<0.001	<0.001	<0.001	
		SF-36 MCS	NS	NS	NS	
		**Group 2: QG + RM**	**After QG**	**After RM**	**F-UP**	
		FIQ	<0.05	<0.001	<0.05	
		HAQ	<0.001	<0.001	<0.001	
		NRS (pain)	<0.001	<0.001	<0.001	
		RPS (pain)	<0.001	<0.001	<0.001	
		NRS (sleep)	NS	<0.05	NS	
		HADS-A	<0.001	<0.001	<0.001	
		HADS-D	<0.001	<0.001	<0.001	
		SF-36 PCS	<0.05	<0.001	<0.001	
		SF-36 MCS	NS	NS	NS	
		Note: (1) QG and RM produce comparable benefits compared to BL over a range of outcomes when applied as an initial intervention. When they are implemented as a sequential intervention, most outcomes are not different from the end of the first intervention. (2) Effects are generally maintained in the follow-up interval to 6 mos. (3) SMD values for FIQ, HAQ, RPS, HADS-A, HADS-D, and SF-36 PCS generally range from 0.7 to 1.5 in both groups (8 wks–6 mos).				

BA, body awareness; BARS, body awareness rating scale; BDI, Beck Depression Inventory; BL, baseline; CG, control or comparison group; del, delayed intervention group; FIQ, fibromyalgia impact questionnaire; FM, fibromyalgia; F-UP, follow-up; HADS, Hospital Anxiety and Depression Scale (A-anxiety, D-depression); HAQ, health assessment questionnaire; hrs, hours; imm, immediate intervention group; MFI, Multidimensional Fatigue Inventory; MM, mindfulness meditation; mos, months; NRS, numerical rating scale; NS, nonsignificant (*P* > 0.05); PSQI, Pittsburgh Sleep Quality Index; QG, qigong group; QOL, quality of life; RM, Rességuier method; RPS Regional Pain Scale; SF-36, short form-36 (MCS mental component summary, PCS physical component summary); SF-MPQ, short form- McGill pain questionnaire; SMD, standard mean difference; STAI, State Anxiety Inventory; T1, treatment 1; T2, treatment 2; VNS, visual numerical scale; wks, weeks; yrs, years.

**Table 2:** Summary of other studies of qigong for fibromyalgia.

Study Participants, features	Intervention, duration	Outcomes
(1) Creamer et al. 2000 [34] Pilot study, open trial *N* = 28 Mean age: 47.9 yrs Attrition: 8/28 (29%) did not complete 5/8 sessions Note: (1) No control group. (2) Data following intervention was also reported by Singh et al. (1998) [35]	Attendance at 8 weekly sessions (2.5 hrs each), with educational and cognitive/behavioural component (30 min), relaxation/meditation (60 min), qigong (60 min, form not specified) Duration: 8 weeks Measures: BL, 8 wks, 4 mos F-UP, 6 mos F-UP	Significant (*P* < 0.05) improvements in FIQ, sleep, patient global, RAND Health Survey (physical function, energy, emotional function, social function, and pain), coping, depression, and function following the intervention. Benefits were generally maintained at 4 and 6 months from BL. Note: (1) Intent-to-treat analysis of results. (2) Due to multiple interventions during the weekly session, it is not possible to ascribe effects specifically to qigong.

(2) Chen et al. 2006 [36] Pilot study, open trial *N* = 13 Mean age: 49.8 yrs Mean FM duration: 6.2 yrs Attrition: 3/13 (23%) dropped out after 1–3 sessions; data for *N* = 10 analysed postintervention; *N* = 8 returned for 3 mos F-UP Note: No control group.	External qigong therapy applied for 5–7 sessions (45 mins) over 3 wks; monthly maintenance session Intervention duration: 3 weeks Measures: BL, 3 wks, 1 mo F-UP, 3 mos F-UP	Significant improvements in impact (FIQ), depression (BDI), pain (MPQ, VAS), and anxiety following the intervention. Changes were maintained at 1 and 3 months, although there was some rebound. Pre-post SMD values were 1.1–1.9 at 3 wks, 0.7–1.7 at the 1 mo F-UP, and 0.8–1.4 at the 3 mos F-UP. Sleep (PSQI) scores were not significantly improved. Note: Two cases had such dramatic and persistent benefit following treatment that they were considered “cured.” Individual outcomes for FIQ, tender points, MPQ, and BDI are presented and indicate minimal post-treatment symptomology.

(3) Lynch et al. 2009 [37] Pilot study, open trial *N* = 23 Mean age: 51.1 yrs Mean FM duration: 12.0 yrs Attrition: 21 (91%) completed 4 wks, 14 (61%) 9 wks, 13 (52%) 3 mos, and 12 (52%) 6 mos. Note: No control group.	Two half-day (4 hrs) qigong training sessions (level 1 CFQ), weekly review/practice session (90 min), daily home-practice (45 min). Intervention duration: 9 wks Measures: BL, 9 wks, 3 mos F-UP, 6 mos F-UP Note: (1) Level 1 CFQ consists of movements. (2) No measure of home-practice was included.	Significant improvements in pain (NRS), impact (FIQ), and physical function (SF-36), either following treatment or at F-UP. Note: (1) Analysis conducted on *N* = 12 who completed the 6- month trial. (2) Pre-post SMD values for pain, impact, and physical function were 0.6–0.9 following the intervention and at follow-up.

(4) Sawynok et al. 2013 [38] Case reports, *N* = 2 Age: #1 = 45 yrs, #2 = 57 yrs FM duration: #1 = 10 yrs, #2 = 20 yrs Note: (1) Both cases had extensive symptomology (pain, disturbed sleep, and multiple other symptoms) that had progressed over time. (2) Both had previously tried many other therapies (pharmacological, nondrug, complementary methods) but remained symptomatic.	Extensive qigong practice (levels 1 and 2 CFQ). Both attended an initial 8-day workshop in 2008 and subsequently practiced daily for an extended interval (≥1 hr for 6 mos); one undertook repeat training/practice sessions. In 2009 and 2010, both attended additional workshops (8–10 days) and continued extensive daily practice (1.5–3 hrs/day) at times. Note: (1) Level 1 CFQ consists of movements, while level 2 CFQ consists of meditative practice. (2) These individuals undertook extensive amounts of self-practice of qigong. Motivation to engage and continue came from initial health benefits experienced.	**Case 1:** initially, less pain, tension, anxiety; over next few months other treatments ceased (antidepressant, massage, chiropractic) and resumed eating foods that she was allergic to; blood pressure normalized. **1 yr F-UP:** all medications and supplements had ceased; tooth and jaw pain resolved; sleep improved. **3 yr F-UP:** no body-wide pain since 1 yr after starting; pain occasional and localized; tension headaches gone; cognition, sleep, fatigue, mood, skin, and circulation all improved; employment resumed. **Case 2:** initially, improved energy and bladder and bowel function; by 6 mos, vast improvement in pain and other symptoms. **1+ yr F-UP:** improvement in vision at 8 mos; resumed full-time work. **3+ yr F-UP:** resumed eating foods that she was allergic to stopped taking supplements, amitriptyline; resumed her life. Note: (1) Only qualitative comments are available. (2) The range of symptoms that resolve with extended qigong practice is of particular interest.

(5) Sawynok et al. 2013 [39] Extension trial, *N* = 20 Some trial completers from RCT [29] participated in a 6-month extension phase. Mean age: 53 yrs Mean FM duration: 11.5 yrs (extracted from RCT) Attrition: 7/20 (35%) withdrew Note: (1) For those completing this trial, 5/13 (38%) had voluntarily undertaken additional qigong training prior to the extension phase. (2) Both quantitative and qualitative outcomes were recorded.	Two half-day (4 hrs) qigong sessions (level 2 CFQ), weekly review/practice session (60 min), daily home-practice (60 min) for 8 wks. Home-practice mix of levels 1 and 2 CFQ. Duration: 8 wks Measures: BL, 8 wks, 4 mos F-UP, 6 mos F-UP Note: (1) Level 2 CFQ consists of meditation instructions. (2) Practice times self-reported at 8 wks and 4 and 6 mos (via checklist of times).	**Quantitative measures:** For *N* = 13 extension trial completers, there were significant reductions in pain (NRS) and impact (FIQ) and improvements in sleep (PSQI) and physical function (SF-36 physical) compared to baseline. Similar quantitative changes observed in those who had voluntarily undertaken additional training (*N* = 5) and those who had not (*N* = 8). The former had milder baseline symptomology than the latter. **Qualitative responses:** These were considered separately for *N* = 5 and *N* = 8 subgroups. Narrative comments noted benefits in pain, sleep, physical and mental function, and quality of life as assessed in quantitative measures in the *N* = 5 subgroup; comments were more tempered for the *N* = 8 subgroup. There were also health benefits in other areas (food allergies, chemical sensitivities, asthma, migraines, blood pressure, and vision) in the *N* = 5 subgroup; several discontinued medications (for migraine, asthma, sleep, and pain); additional benefits were not noted by the *N* = 8 subgroup. Note: (1) The *N* = 5 subgroup reported the highest home-practice times at 8 wks (~60 min/day), and this was maintained to 6 mos. The *N* = 8 subgroup had lower home-practice times at 8 wks (~40 min/day), and this faded over time (~20 min/day). (2) For some in the *N* = 5 subgroup, open-ended comments indicated home-practice times of 10–15 hrs/week at some stages.

(6)Sawynok and Lynch 2014 [40] Retrospective qualitative analysis of *N* = 73 comments by participants who completed the RCT [29]. Note: Analysis considered (a) narrative comments of extension trial completers versus noncompleters and (b) thematic comments by those who practiced per protocol, minimally, or an intermediate amount.	No additional qigong practice. Retrospective analysis of initial 6- month experience with practice of level 1 CFQ. Post hoc consideration clustered around (a) motivation/perseverance and (b) amount of practice. Note: Per protocol practice: >5 hrs/wk Minimal practice: <3 hrs/wk Intermediate practice: >3 <5 hrs/wk	**Narrative comments:** There was a difference in initial qigong experiences by those who completed the extension trial (*N* = 13) versus those who did not complete the extension (*N* = 7), with more favourable health effects reported by completers. Comments recapitulate quantitative measures but also cover other areas. **Thematic comments:** There was a clear difference in comments on pain, sleep, and quality of life, by those who practiced per protocol versus those who practiced minimally, and these reflect quantitative differences between the two groups. Those who practiced an intermediate amount also had positive comments on experiences, but there was a difference in tone (more moderated) compared to those who practiced per protocol.

BDI, Beck Depression Inventory; BL, baseline; CFQ, Chaoyi Fanhuan Qigong; FIQ, fibromyalgia impact questionnaire; FM, fibromyalgia; F-UP, follow-up; hrs, hours; mo, month; min, minute; MPQ McGill pain questionnaire; NRS, Numerical Rating Scale; PSQI, Pittsburgh Sleep Quality Index; RCT, randomized controlled trial; SF-36, Short Form-36; SMD, standard mean difference; VAS, Visual Analog Scale; wk(s), week(s); yrs, years.

**Table 3:** Methodological issues relating to qigong studies for fibromyalgia.

Issue	Comments
(1) Plurality of forms	(i) Qigong has a long history and is part of traditional Chinese medicine; many forms have evolved; different contexts (martial arts, health benefits, and spiritual development) emphasize different elements. (ii) Qigong is used either generically to refer to collective practices with common elements or specifically to refer to a particular form of practice.

(2) Components of practice	(i) Qigong consists of movement, breath instruction, and mental/mind components. (ii) Qigong is now characterized as “meditative movement” and “mindful exercise,” recognizing a unique form of movement. (iii) Qigong movements emphasize softness, looseness, flowing, and relaxation; this differs from the emphasis on muscular strength or flexibility or aerobic capacity that is part of other exercise regimens.

(3) Amount of practice	(i) Internal qigong involves self-practice, while external qigong involves highly skilled practitioners; most FM studies use self-practice. (ii) Minimal amounts of practice required (threshold for effect) and relationship of amount of practice to outcomes (practice-response relationship) are important. (iii) Threshold requirements can be explored in RCTs where the amount of practice is specified, and adherence to practice is monitored. (iv) The practice-response relationship can be explored in a prospective or retrospective manner within RCTs. However, as the amount of practice increases, there is increased attrition, and retrospective analyses are limited by self-selection. (v) Observational studies of those who engage in extended practice in a controlled setting, or voluntarily in a community setting, provide important information relating to the health potential of qigong.

(4) Effectiveness of practice	Not all practice time is equally effective. Movements can take time to learn, and nuances of execution can matter. With mental instructions, it can be a challenge to determine parameters that reflect effective engagement.

(5) Control/comparator group	(i) Some trials strive to isolate an active component of an intervention, to delineate “specific” from “nonspecific” factors. (ii) With complex interventions, many elements (specific, nonspecific) can be contributive, and isolating specific components can be a challenge. (iii) Control/comparison groups for FM trials can consist of (1) wait-list subjects; (2) sham group (some elements of practice engaged, e.g., movement without mental instruction); (3) education/social support group; (4) active comparator group (e.g., qigong compared to exercise or meditation).

(6) Multiple trial designs	(i) RCTs involve controlled settings, defined inclusion-exclusion criteria, predetermined primary and secondary outcomes, and comparison to placebo or comparator groups, and provide information that is relevant to the regulatory approval process. (ii) Pragmatic trials provide “in situ” information, include more heterogeneous patient groups, compare outcomes to usual care or standards of care, include long-term follow-ups, and provide information that is relevant to clinical practice. (iii) Qualitative research provides further valuable insights. (iv) It is impossible to blind qigong practice, and multiple trial designs are needed to explore efficacy.

(7) Participants, subgroups	(i) FM involves widespread pain and multiple somatic symptoms (disturbances in sleep, mood, and other functions) and can differ in terms of chronicity (duration) and additional symptomology (part of FM or comorbidity). (ii) Post hoc analyses based on demographics or chronicity are possible in larger trials. (iii) Prospective subgroups based on psychological characteristics, motivation, locus of control, and attitudes towards complementary and alternative therapies need to be considered.

(8) Outcomes	(i) FM outcomes can be considered in the context of chronic pain or as condition-specific outcomes. (ii) Comparisons can be between groups (compared to control or comparator) or within groups (compared to baseline). (iii) With the latter approach, it is possible to consider outcomes in relation to those considered to be clinically significant, which have been defined by clinicians and patients. (iv) For a condition with multiple symptoms, an intervention that provides benefits in multiple core domains (pain, sleep, mood, impact, and quality of life) will be of particular relevance for clinical practice.
